# Automated Condition-Based Suppression of the CPR Artifact in ECG Data to Make a Reliable Shock Decision for AEDs during CPR

**DOI:** 10.3390/s21248210

**Published:** 2021-12-08

**Authors:** Shirin Hajeb-Mohammadalipour, Alicia Cascella, Matt Valentine, Ki H. Chon

**Affiliations:** 1 Biomedical Engineering Department, University of Connecticut, Storrs, CT 06269, USA; ki.chon@uconn.edu; 2 Defibtech, LLC, Guilford, CT 06437, USA; acascella@defibtech.com (A.C.); mvalentine@defibtech.com (M.V.)

**Keywords:** chest compression, CPR, AED, ECG, shockable, non-shockable, cardiac arrest

## Abstract

Cardiopulmonary resuscitation (CPR) corrupts the morphology of the electrocardiogram (ECG) signal, resulting in an inaccurate automated external defibrillator (AED) rhythm analysis. Consequently, most current AEDs prohibit CPR during the rhythm analysis period, thereby decreasing the survival rate. To overcome this limitation, we designed a condition-based filtering algorithm that consists of three stop-band filters which are turned either ‘on’ or ‘off’ depending on the ECG’s spectral characteristics. Typically, removing the artifact’s higher frequency peaks in addition to the highest frequency peak eliminates most of the ECG’s morphological disturbance on the non-shockable rhythms. However, the shockable rhythms usually have dynamics in the frequency range of (3–6) Hz, which in certain cases coincide with CPR compression’s harmonic frequencies, hence, removing them may lead to destruction of the shockable signal’s dynamics. The proposed algorithm achieves CPR artifact removal without compromising the integrity of the shockable rhythm by considering three different spectral factors. The dataset from the PhysioNet archive was used to develop this condition-based approach. To quantify the performance of the approach on a separate dataset, three performance metrics were computed: the correlation coefficient, signal-to-noise ratio (SNR), and accuracy of Defibtech’s shock decision algorithm. This dataset, containing 14 s ECG segments of different types of rhythms from 458 subjects, belongs to Defibtech commercial AED’s validation set. The CPR artifact data from 52 different resuscitators were added to artifact-free ECG data to create 23,816 CPR-contaminated data segments. From this, 82% of the filtered shockable and 70% of the filtered non-shockable ECG data were highly correlated (>0.7) with the artifact-free ECG; this value was only 13 and 12% for CPR-contaminated shockable and non-shockable, respectively, without our filtering approach. The SNR improvement was 4.5 ± 2.5 dB, averaging over the entire dataset. Defibtech’s rhythm analysis algorithm was applied to the filtered data. We found a sensitivity improvement from 67.7 to 91.3% and 62.7 to 78% for VF and rapid VT, respectively, and specificity improved from 96.2 to 96.5% and 91.5 to 92.7% for normal sinus rhythm (NSR) and other non-shockables, respectively.

## 1. Introduction

Out of hospital cardiac arrest (OHCA) affects more than 325,000 people in the United States each year. This occurs either due to shockable rhythms, such as rapid ventricular tachycardia (RVT) and ventricular fibrillation (VF), or non-shockable rhythms such as asystole and pulseless electrical activity (PEA) [1]. Two-thirds of OHCAs start as a non-shockable rhythm [2]. The most effective treatment for non-shockable rhythms is cardiopulmonary resuscitation (CPR). For shockable RVT and VF, applying electrical shock with an automated external defibrillator (AED) in conjunction with CPR is critical to reset heart activity, according to the American Heart Association’s (AHA) 2020 guidelines [3]. Most AEDs’ algorithms automatically make shock versus no-shock decisions. However, during CPR, chest compressions (CCs) induce severe artifacts in ECG that can destroy the morphology of the waveforms. CPR artifact on shockable rhythms can resemble a regular rhythm with a rate equal to CCs. Consequently, an AED’s arrhythmia detection algorithm may be fooled by the rhythmicity of the CPR artifact and make an incorrect non-shock decision for a shockable rhythm. Conversely, CCs may also add fast and disorganized components to non-shockable rhythms which may lead to misclassification as shockable. Hence, to reduce inaccurate shock versus non-shock classification, most current AEDs require preshock CPR interruptions to make reliable rhythm analyses [4,5,6]. These interruptions increase the severity of the ischemic injury both to the heart and to the brain. Moreover, resuming CPR after interruption does not promptly lead to the full restoration of forward blood flow [7]. Consequently, CPR interruptions reduce the survival rate of OHCA patients. 

Various filtering methods using numerous signal processing techniques have been developed during the last two decades to suppress CPR artifacts [5,6,8]. The majority of studies are based on Kalman filters [9,10], different adaptive filtering methods such as least mean square (LMS) [11,12], the enhanced adaptive method [13,14], and recursive least squares (RLS) [15,16]. Almost all of the above-noted methods require one or more reference signals (such as chest pressure, chest displacement, chest acceleration, compression depth, or thoracic impedance). As most of the current AEDs do not have the hardware availability to capture the reference signal, the dependency on the reference signal is a deficiency of the available methods [17]. Unfortunately, only a few algorithms have been developed without reference signals. Therefore, despite all the complicated filtering methods that have been introduced in the literature, the sensitivity (SE) and specificity (SP) of AED rhythm analysis need to be further improved in the absence of a reference signal. 

In this study, we aimed to provide a condition-based filtering approach that could effectively work for both shockable and non-shockable rhythms using only ECG data. In all past studies, major efforts to suppress CPR artifact, in the absence of a reference signal, involved filtering only the fundamental frequency. However, the chest compression rate is not perfectly sinusoidal, hence, harmonic frequency peaks are present, and they consequently obfuscate ECG morphology. Hence, to address this issue, we propose a condition-based filtering algorithm which not only suppresses the fundamental frequency component of the CPR artifact but, depending on the type of rhythm (e.g., non-shockable), harmonics associated with CPR artifacts are also removed. However, the spectral peaks associated with the harmonics of the CPR artifacts are not removed for shockable rhythms as their frequency content overlaps with the dynamics of the shockable signal. The question is then how to determine if the CPR artifact-contaminated signal is shockable or non-shockable. This is a difficult problem, as the whole premise of filtering is to remove as much of the CPR artifact as possible but knowing, a priori, the type of rhythm (shockable or non-shockable) is difficult and yet the key problem to solve. To address this issue, we implemented three different stop-band filters and they are used when predefined conditions are met. These condition-based filter criteria were derived from diverse and extensive development datasets consisting of the PhysioNet Physio bank archive and CPR artifact from Defibtech commercial AED log files. We tested the proposed condition-based filtering approach on an independent test dataset. Details of the filtering algorithm approaches based on different conditions are provided in the next section.

## 2. Materials and Methods

### 2.1. ECG Databases

Several different data sources have been used in this study to create development and separate validation data sets:

#### 2.1.1. Development Set

To create our development data set, ECG recordings from the PhysioNet Physiobank online archive were used, including Massachusetts Institute of Technology–Beth Israel hospital (MIT–BIH) malignant ventricular arrhythmia database (VFDB) [18], Creighton University tachyarrhythmia database (CUDB) [19], and the sudden cardiac death Holter database (SDDB) [18]. All these recordings have a sampling frequency of 250 Hz with 12-bit resolution over a 10-mV range. Using annotation files, 50 subjects’ non-shockable and 45 subjects’ shockable ECG rhythms were used. All recordings were resampled at a sampling rate of 125 Hz. We only used 14 s samples of each subject’s data. CPR-contaminated ECG data were created by adding CPR artifacts to the clean ECG rhythms using the following equation [20]:

(1) $$ECG_{Corrupted}=ECG_{Clean}+\left[std\;(ECG_{Clean}\right)\times 10^{\left(\frac{-SNR}{20}\right)}\times \frac{CPR}{std\left(CPR\right)}]$$

It is based on the assumption that CPR artifact is an additive noise independent of the underlying ECG. In this equation the terms $ECG_{Clean}$, $ECG_{Corrupted}$, and CPR stand for artifact-free ECG, CPR-contaminated ECG, and CPR artifact, respectively. Fifty-two different CPR performers created their own unique signature of CPR artifacts, and they were added to every 14 s ECG segment to create 2340 shockable and 2600 non-shockable CPR-contaminated ECG data segments. The signal-to-noise ratio (SNR) of the development dataset was set to −3 dB These samples were used in the development phase to design our filtering algorithm, and the decision process on when to use certain types of filters. 

#### 2.1.2. Separate Validation Set

ECG recordings prepared by Defibtech were used as an independent validation set to evaluate the algorithm’s performance. This dataset contained 396 subjects’ non-shockable rhythms including normal sinus rhythm (NSR), supra VT, rapid supra VT, sinus bradycardia, atrial fibrillation (AF), atrial flutter (AFL), heart block, and premature ventricular contractions (PVCs), and 72 shockable subjects including rapid VT and VF. The AEDs’ sampling frequency was 125 Hz. In total, we created 20,592 non-shockable, and 3744 shockable CPR-contaminated ECG data. 

**Figure 1:**
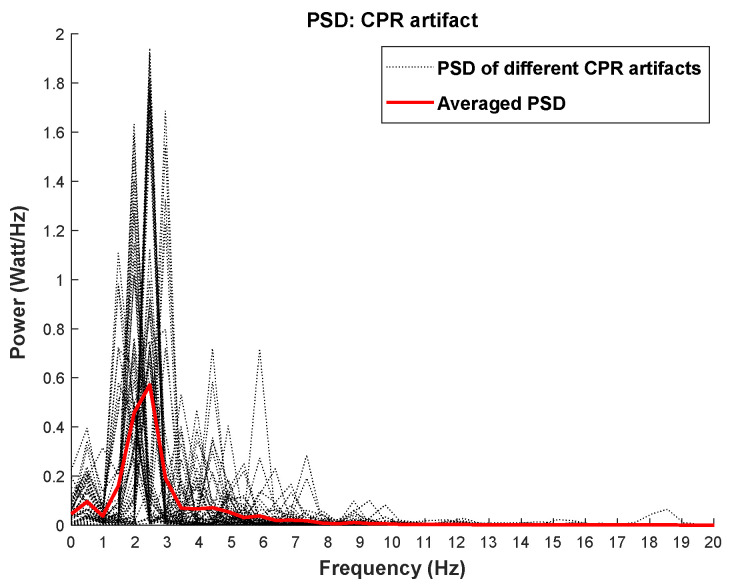
PSD of different CPR artifacts and the averaged PSD are shown in black dashed lines and red line, respectively.

All human-induced CPR artifacts were obtained from Defibtech AED devices during asystole. Since there is no cardiac contraction during asystole, any electrical activity in recorded ECG during the CPR process can be considered as a true CPR artifact. The artifacts used in this study are from 52 different resuscitators. The SNR of the CPR-contaminated ECG was set to −3 dB. In Figure 1, black dashed lines indicate the power spectral density (PSD) of each CPR artifact and the red line shows the averaged PSD of all artifact samples. As shown in Figure 1, CPR artifacts are diverse in terms of compression depths and rates. Different SNRs were also used to create a validation set with multiple corruption levels. Details will be discussed in the Results section. 

### 2.2. The Condition-Based Filter

**Figure 2:**
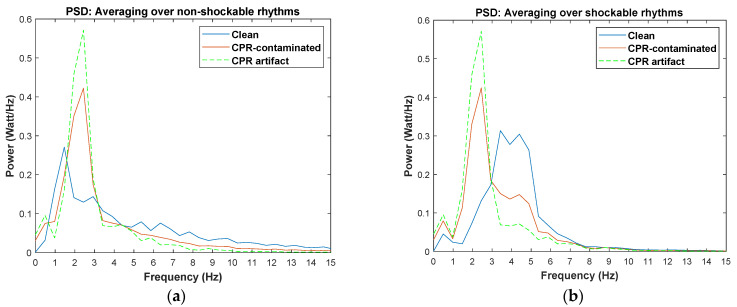
Averaged PSD of (**a**) non-shockable and (**b**) shockable ECG rhythms. In both figures, blue and red represent the PSD of clean ECG and CPR-contaminated ECG, respectively.

**Figure 3:**
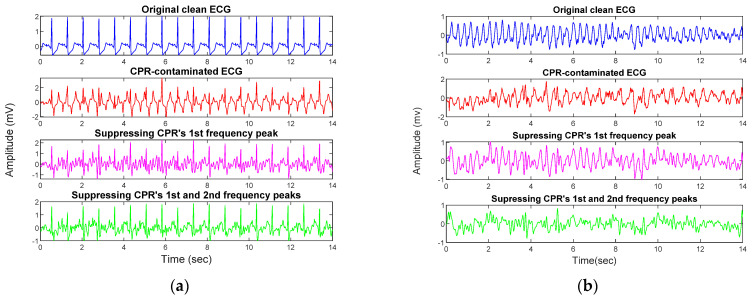

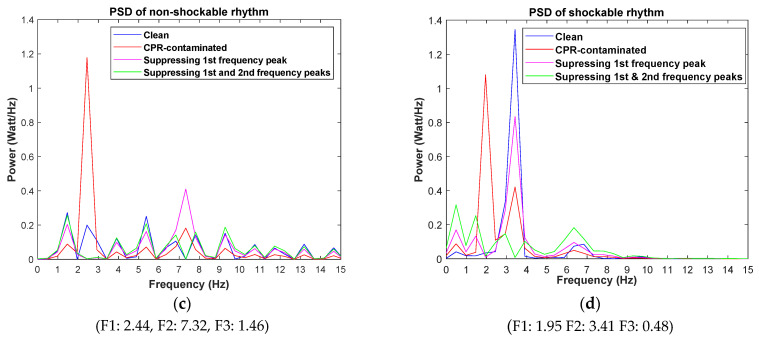
(**a**) Non-shockable ECG sample and (**b**) shockable ECG sample—1st row: desired clean ECG, 2nd row: CPR-contaminated ECG, 3rd row: filtered ECG after removing highest frequency peak, 4th row: filtered ECG after removing both highest and 2nd frequency peaks. (**c**) PSD plots of non-shockable ECG sample and (**d**) PSD plots of shockable ECG sample. The PSD of the desired clean ECG, CPR-contaminated ECG, filtered ECG after removing highest frequency peak, and filtered ECG after removing both highest and 2nd frequency peaks are plotted in blue, red, purple, and green, respectively.

The condition-based filtering algorithm contains three stop-band filters. The status of each filter can be active or non-active depending on the output of the defined condition. The cutoff frequencies of the filters are not fixed but are updated according to the characteristics of the ECG data segment. In order to develop the condition-based approach, different characteristics of the shockable and non-shockable rhythms as well as the features of the CPR artifacts have been inspected and characterized in the development phase. Consequently, the development dataset was used to define proper conditions based on the following factors:

- To suppress the CPR components from the underlying ECG time series, the dominant frequency of CPR artifact needs to be detected and removed. According to [21], the recommended rate for performing chest compressions during CPR is 100–120 compressions per minute. However, CPR’s fundamental frequency can be observed as high as 3 Hz when performed too quickly. PSD plots of different CPR artifact samples for both shockable and non-shockable rhythms shown in Figure 1 demonstrate that the fundamental frequency of the chest compressions is localized within (1–3) Hz. In this figure, the red line is the averaged PSD of all CPR samples.
- It is widely recognized that the spectral content of non-shockable ECG rhythms is distributed over a wider frequency band than that of shockable rhythms (Figure 2a) [5]. The shockable rhythms’ spectral power is largely concentrated within (3–6) Hz, as reported in [12,22], and also shown in Figure 2b. In Figure 2, the averaged PSD for both non-shockable and shockable rhythm types are shown in the left and right panels, respectively. PSDs of both rhythm types are similar when they are contaminated with CPR artifact (red lines in Figure 2a,b).
- For most non-shockable rhythms, removing CPR harmonic peaks in addition to the CPR fundamental frequency results in the recovery of the uncontaminated ECG data, but this is not always the case for shockable rhythms. According to our comprehensive analysis in the development phase, the best filtering approach would remove artifacts’ first- and second-largest spectral peaks for non-shockables and only the largest frequency peak for shockables (unless the largest spectral peak does not appear within (3–6) Hz). Figure 3 shows representative examples for CPR-contaminated non-shockable and shockable rhythms after removing artifact frequency peaks. However, in real-life scenarios, this decision process is not feasible because the type of underlying rhythm is unknown. To solve this issue, we propose a solution that defines a set of conditions to avoid shockable rhythm distortion as much as possible.
- If the artifact’s largest spectral peak is below 1.5 Hz, the first harmonic spectral peak would not fall close to shockable samples’ dominant frequency ((3–6) Hz). Therefore, the algorithm removes this harmonic component.
- Non-shockable samples have spectral power in the higher frequency bands (above 10 Hz) whereas the shockable samples do not share this characteristic [12]. After suppressing the frequency of the largest spectral peak of the CPR artifact for non-shockable data, the total power within (10–15) Hz is determined; it is then used to determine whether or not a second filter is needed to remove the second largest spectral peak.

**Figure 4:**
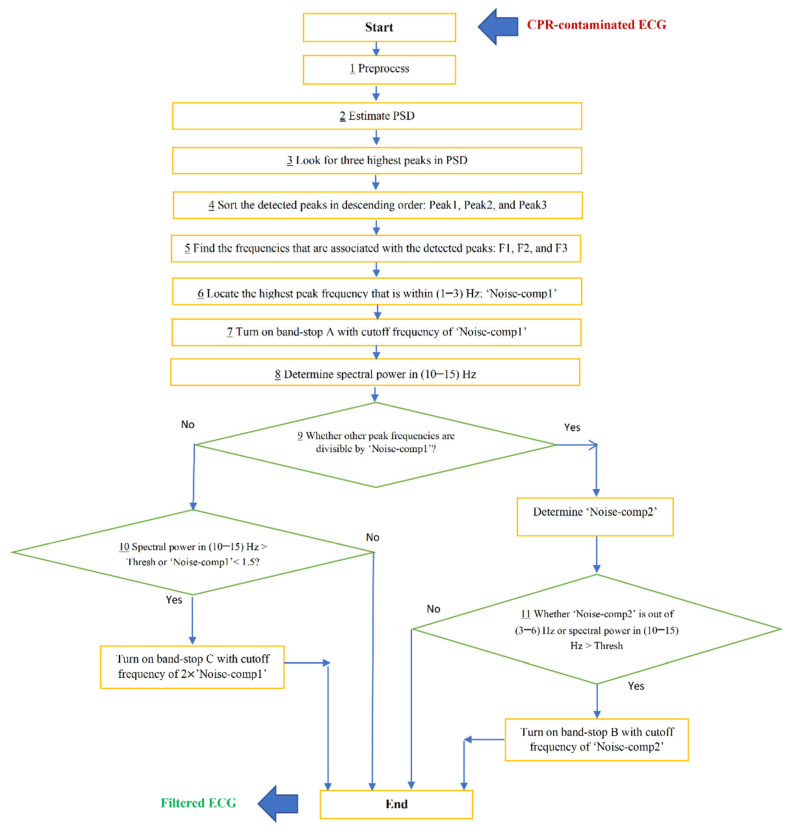
Block diagram of the proposed filtering algorithm.

Figure 4 shows the block diagram of the condition-based filtering algorithm we designed. The above-noted process is succinctly summarized as follows:

1. Preprocess ECG data segment by applying a 2nd order infinite impulse response (IIR) notch filter to remove 60 Hz electrical noise; also apply wavelet implementation plus averaging to remove glitches and achieve a smoother signal. Use Daubechies 6-tap wavelet to perform signal decomposition at 10 different levels. Subsequently, subtract the reconstructed approximation signal from the original signal.
2. Estimate PSD using Welch’s overlapped segment averaging spectral estimator. Use the Hamming window. Average the modified periodograms to obtain the PSD estimate.
3. Look for the three highest peaks in the PSD of the CPR-contaminated ECG data segment.
4. Sort the detected peaks in descending order: Peak 1, Peak 2, and Peak 3.
5. Find the frequencies that are associated with each of the detected spectral peaks: F1, F2, and F3.
6. Find which of the F1, F2, and F3 are within (1–3) Hz and label this frequency as Noise-comp1.
7. Turn on the first stop-band filter with the cutoff frequency of Noise-comp1.
8. Determine the spectral power in the frequency band (10–15 Hz) of the resulting filtered signal.
9. Evaluate condition 1: If any of the remaining frequency peaks are divisible by Noise-comp1, then define Noise-comp2 (in case both are divisible, select the frequency component with the highest power), else skip step 10 and move forward to step 11.
10. Evaluate condition 2: If Noise-comp2 is not within (3–6) Hz or spectral power within (10–15) Hz satisfies the threshold value, then turn on stop-band 2 with the cutoff frequency of Noise-comp2. -> END. (Details on the derivation of the spectral power value threshold are provided in the Development Phase of the Results section below).
11. Evaluate condition 3: If spectral power within (10–15) Hz satisfies the threshold value or Noise-comp1 is less than 1.5, then turn on stop-band 3 with cutoff frequency of 2* Noise-comp1. -> END.

In addition to the proposed filtering method, we have also implemented and tested the machine learning-based algorithm that was introduced in [23]. This approach is based on extracting 21 features from the CPR-contaminated ECG signal. Subsequently, only 13 significant features were selected to train the back propagation (BP) neural network classifier. More detail about the type of features as well as the feature selection method can be found in the original study [23]. This algorithm is considered as the category of approaches that directly analyzes and classifies CPR-contaminated ECGs with no prefiltering stage. Given the promising results via BP neural network for shock versus no-shock classification (SE and SP > 99%) on unfiltered ECG, we used ECG recordings from our validation dataset to examine our condition-based filtering technique combined with the BP neural network method.

## 3. Results

This section includes results of the development phase and testing phase. 

### 3.1. Development Phase

During the development process, a development dataset was analyzed in order to design the proposed filtering algorithm. Our development set contains different types of non-shockable and shockable rhythms from 50 and 45 different subjects, respectively. This dataset is diverse, as ECG recordings were acquired from three different databases: CUDB, VFDB, and SDDB. The results of suppressing the CPR artifacts’ first and second largest spectral peaks in the non-shockable and shockable ECG rhythms are shown in the left and right columns of Figure 3, respectively. The first, second, third, and fourth rows of Figure 3a,b represent the artifact-free ECG, CPR-contaminated ECG, filtered ECG after removing the artifacts’ highest spectral peak, and filtered ECG after removing the artifacts’ first and second largest spectral peaks, respectively. The PSD of the data in Figure 3a,b are plotted for the artifact-free ECG (blue lines), CPR-contaminated ECG (red lines), ECG after removal of the highest spectral peak (purple lines), and ECG after removal of the highest (2.44 Hz) and the second largest spectral (7.32 Hz) peaks (in green) in Figure 3c,d. Comparing artifact-free ECG with filtered ECGs, it is shown that, in the case of non-shockable rhythm, the PSD of the ECG after removing the two largest spectral peaks is most correlated with the PSD of the non-shockable artifact-free ECG (compare blue and green lines in Figure 3c as well as the bottom vs. the first row in Figure 3a). However, in the case of shockable rhythms, removing the artifacts’ second highest spectral peak (3.41 Hz) appears to be detrimental, as it introduced spectral distortion. This is because the fundamental frequency component of the shockable rhythms overlapped with the harmonic frequencies of the chest compressions. Figure 3d demonstrates that in the case of shockable ECG, the highest PSD correlation is achieved between ECG after removing only the highest spectral peak (purple) and the artifact-free ECG (blue). 

Table 1 shows Pearson’s correlation coefficient values between PSDs of artifact-free ECGs and filtered ECGs for both shockable (45 subjects) and non-shockable (50 subjects) rhythms for the development dataset. Our analysis shows that 64% of the artifact-free non-shockable rhythms are highly correlated (>0.7) with the filtered signal when we remove only the highest spectral peak of the artifact. However, this value is increased up to nearly 76% when we remove the two largest spectral peaks. However, this observation is invalid for the shockable rhythms. By removing only the main frequency component of the artifact, 78% of filtered samples are highly correlated with the artifact-free ECG. However, this correlation value drops to 66% by removing the second highest spectral peak. The proposed condition-based algorithm can solve this issue. 

**Figure 5:**
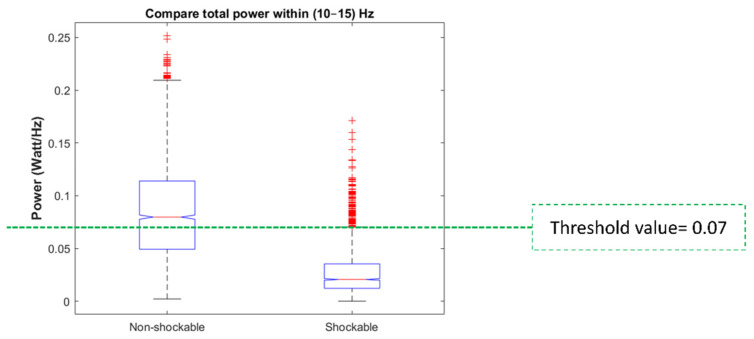
Box plots for comparing spectral power in the frequency band (10–15) Hz for shockable and non-shockable samples for the development set.

To determine the threshold value for the spectral power in the frequency band (10–15) Hz, their boxplots are plotted for both shockable and non-shockable samples (Figure 5). Mean standard deviation values are also shown in Table 2. According to Figure 5 the upper limit of the spectral power in the frequency band (10–15) Hz for shockable samples is 0.072. For example, only 6% of shockable samples have spectral power above 0.072 and they are the outliers (shown as “+” marks). Therefore, a threshold value of 0.07 Watt/Hz was set to avoid removing the artifacts’ second highest spectral peak from the shockable rhythms. 

### 3.2. Testing Phase

**Figure 6:**
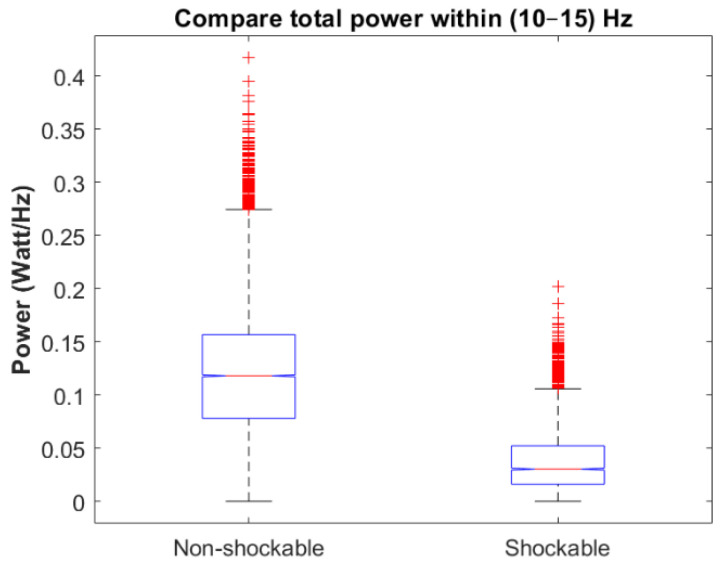
Box plots for comparing spectral power in the frequency band (10–15) Hz for shockable and non-shockable samples for the validation set.

**Figure 7:**
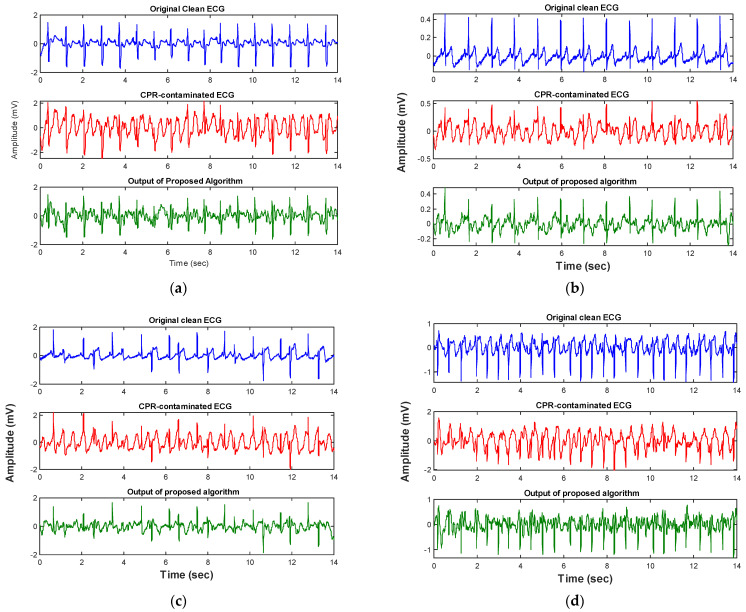
(**a**–**d**) Show the performance of the condition-based filter on four different representative non-shockable ECG rhythm types (test set). Each panel represents the ECG data segment of a different subject.

**Figure 8:**
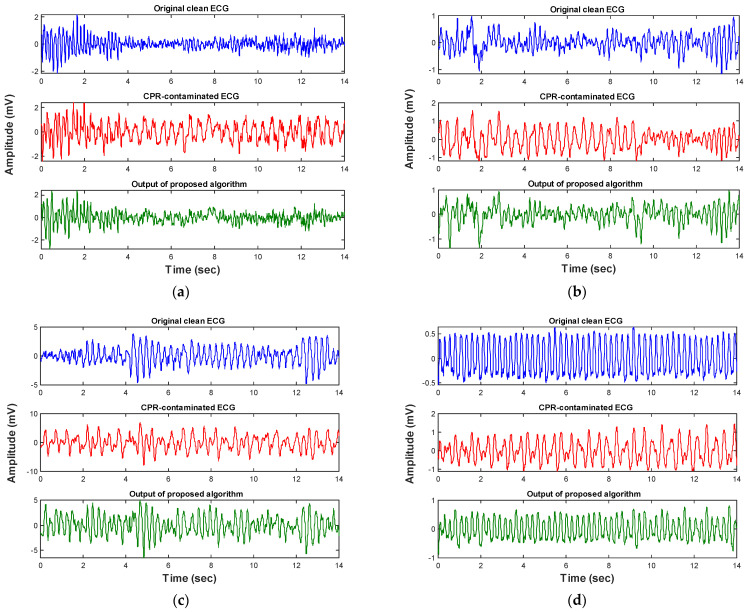
(**a**–**d**) Show the performance of the condition-based filter on four different representative shockable ECG rhythm types (test set). Each panel represents the ECG data segment of a different subject.

The performance of the algorithm was further evaluated on an independent validation data set. Figure 6 and Table 3 indicate that the defined threshold value for the spectral power in the frequency band (10–15) Hz is also valid for the unseen ECG data. Our analysis showed that nearly 80% of the non-shockable samples have total spectral power higher than the defined threshold value while only 12% of the shockables breach this threshold. Figure 7 and Figure 8 show representative results of applying the condition-based algorithm on four different shockable and non-shockable data segments. In order to quantify the performance of the proposed condition-based filtering algorithm on the validation data set, three different criteria have been evaluated: (1) SNR improvement, (2) correlation coefficient, and (3) results of the AED’s rhythm classification algorithm. 

Using the proposed algorithm, the SNR value improved for all rhythm types (Table 4). The average SNR improvement was 4.8 dB for NSR and 4.5 dB for other rhythm types of non-shockables. In the case of shockables, the SNR improvement was 4.08 dB for VF and 3.68 dB for fast VT. 

Computing the correlation coefficient between the PSD of the artifact-free ECG and the filtered ECG, 70% of the filtered non-shockable and 82% of filtered shockable data were highly correlated (>0.7) with the artifact-free ECG. The correlation value greater than 0.7 was only about 12% and 13% between the CPR-contaminated ECG and the corresponding artifact-free ECG for non-shockables and shockables, respectively. These values are shown in the fourth column of Table 4 for each rhythm category. 

The third performance evaluation metric was the determination of shockable versus non-shockable classifications. The last column of Table 4 represents the results of applying our filtering method on the validation set followed by the Defibtech AED’s classification algorithm. It should be noted that, Defibtech’s shock decision algorithm uses standard ECG processing techniques similar to other commercial AEDs. These techniques as well as the algorithm performance of the different commercial AEDs have been previously described in [24,25,26]. Comparing the values in the last column with column 4, the proposed filtering method improved the classification outcome, as the SE of the shockable rhythm detection for VF and rapid VT increased from 67.7 to 91.3%, and from 62.7 to 78%, respectively. These improved SE values of shockable rhythm detection satisfy the American Heart Association’s AED requirement [27].

**Figure 9:**
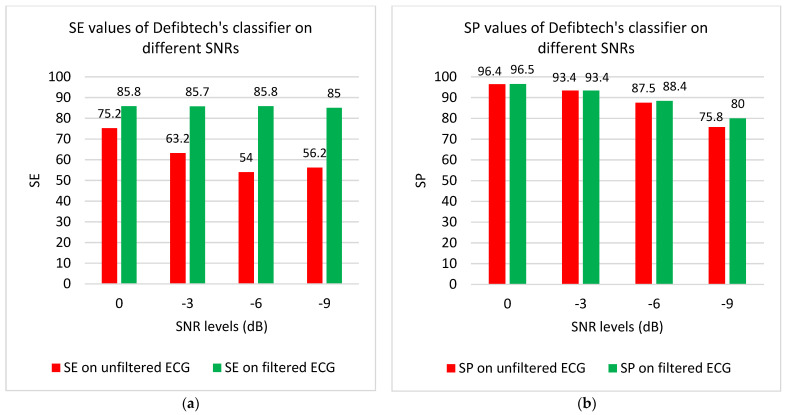

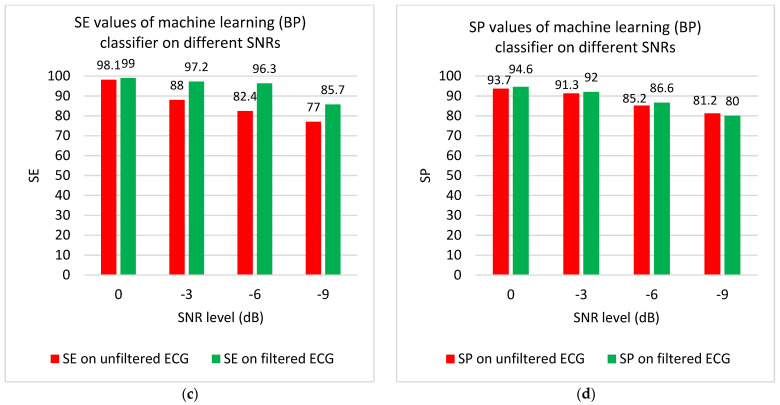
(**a**–**d**) represent results of two rhythm analysis algorithms on different corruption levels before and after applying the proposed filtering approach.

Since the true SNR level of the CPR artifact is not known, we also evaluated the performance of the proposed algorithm on four different SNR levels. Figure 9a,b represents the SE and SP values before and after applying the proposed filter on different SNR levels including 0, −3, −6, and −9 dB. As shown, there is a notable SE improvement for all SNR levels when our proposed filtering algorithm is applied. For example, for an SNR of −9 dB (which can be considered as a severe corruption level [28]) the SE increased from 56.2% to 85% (see Figure 9a). For both pre- and postfiltering approaches, the differences in their performances for SP values for all SNR were negligible, albeit they all show decreased values with decreasing SNR (see Figure 9b). Cases 1–2 in Table 5 demonstrate the averaged SE, SP, and ACC values for all corruption levels for applying Defibtech’s AED classification algorithm without and with our proposed filtering algorithm, respectively. 

In order to further represent the impact of our condition-based filtering method in improving the results of rhythm analysis algorithms, we also combined the proposed method with the backpropagation (BP) neural network approach introduced in [23] for shockable vs. non-shockable classification. Results of this combined approach are shown in Figure 9c,d and the cases 3–4 in Table 5. All ECG samples were split into training and test datasets. The 52 CPR artifact samples were also divided randomly into two parts, having 26 CPR artifact samples for both training and test datasets. These CPR-contaminated samples were added to the clean data so that different SNR levels of 0, −3, −6, and −9 dB were achieved. Consequently, the training (with 4914 non-shockable and 910 shockable samples) and test (with 5122 non-shockable and 962 shockable samples) datasets were created. A weighting technique was used to make a more balanced training dataset. Using this technique, a higher error weight was assigned to the class with the lower number of samples [23]. Overall training and testing processes were repeated five times and the results were then averaged. Figure 9 and Table 4 demonstrate the SE values for shockable and SP values for non-shockable rhythms. According to panel c of Figure 9, when our proposed filtering algorithm was combined with the BP neural network classification algorithm, the higher SE values (>96%) were achieved. More importantly, this is still true for low SNR level of −6 dB, as well. As expected, SE values decrease with lower SNR levels. However, the filtering approach performed well despite severe corruption levels as SE increased from 77 to 85.7% for SNR level of −9 dB (see Figure 9c). As shown in Table 5, the averaged SE, SP, and the overall accuracy (ACC) of the BP classifier for all SNR corruption levels are 86.3, 87.8, and 87.6%, respectively; applying the proposed filtering approach increased these values to 94.5, 88.3, and 89.2%, respectively. The results in Table 5 indicate that when our proposed filtering algorithm (fourth row) was combined with the BP neural network classification algorithm instead of Defibtech’s classification algorithm, this combined approach provided the best results in shockable rhythm detection with the averaged SE value of 94.5% for all SNR corruption levels. However, the Defibtech classifier has a slightly better performance for non-shockable rhythm detection. 

## 4. Discussion

This paper introduced a condition-based filtering method to remove CPR motion artifact using a series of stop-band filters. We investigated PSDs of the clean and corrupted shockable and non-shockable rhythms to create filters that perform efficiently for different types of ECG rhythms. Our algorithm was effective in suppressing the CPR artifact from ECG data. Depending on the type of rhythm, the method is designed to filter only CPR artifact without removing the dynamics of the signal of interest. This was accomplished by the algorithm checking three different conditions to decide whether to suppress the PSD’s second largest spectral peak in addition to the first highest spectral peak (the artifacts’ fundamental frequency) of the CPR-contaminated ECG data. The condition-based filter approach as well as the choice of a threshold value for the total spectral power in the frequency range of (10–15) Hz were derived from a development dataset and tested with an independent validation dataset. 

The validation data results demonstrate that the proposed condition-based filter was effective in suppressing CPR artifacts, which led to improved accuracy of AED rhythm analysis when compared to without the use of the filters. Our filtering method does not use any reference signals to filter CPR artifacts. Hence, a comparison to methods that do is not provided. However, it is likely that most methods that use a reference signal may provide better results than provided with the proposed work, albeit with higher computational complexity. To increase survival after cardiac arrest, the next generation of AEDs require real-time rhythm analysis without halting CPR, hence, a simple and effective algorithm is needed [6]. Our algorithm’s implementation in MATLAB 2021a on a Dell XP workstation using a 14 s data segment takes only 90 to 168 msec depending on the number of times a band-stop filter is activated. Hence, with further optimization, certainly, the proposed algorithm is real-time realizable. We were not able to find any published reports stating the computational time of other methods, but most mentioned that the reference-based algorithms were computationally intensive.

Filtering methods based on removing the frequency associated with the CPR artifact have been introduced in the past [5,29,30,31]. For example, in [29], the objective was to design an algorithm to suppress CPR artifacts on VF cases. The algorithm consisted of a single stop-band filter that adapted to the fundamental frequency of the CPR artifact which was obtained from the spectral analysis of the ECG signal. They were able to improve only the SE of VF detection by having the designed filter prior to the shock advice algorithm. However, they did not evaluate their algorithm’s efficacy on shockable VT as well as non-shockable rhythms. One of the first studies dealing with CPR artifacts using only ECG data was published in 2008 [30]. In [30], a spectral filtering approach to remove CPR artifacts in both shockable and non-shockable rhythms was used. In this work, the CPR artifact was modeled as the fundamental frequency of the chest compressions via spectral analysis of the ECG. The first harmonic was only considered in the case of low CC rates (<1.9 Hz). Kalman filtering was then used to suppress the modeled CPR artifacts. The SE improved after applying the filtering method, but the SP decreased by 18.7 points for asystole and by 11 points for all other types of non-shockable rhythms. Subsequently, in 2012, a method based on wavelet analysis and morphology consistency was introduced to detect VF rhythm types [32]. They achieved SE of 91% and SP of 85%. The main limitation of such techniques is that the algorithm was effective mostly on NSR but it was less accurate for PVC beats or other types of ectopic beats [33]. In 2016 [34], a new filtering method was introduced based on adaptively incorporating noise-assisted multivariate empirical mode decomposition to estimate the model of CPR artifacts in VF rhythms. Subsequently, back propagation (BP) neural network (NN) was used to perform the shock/non-shock decision [34]. The authors used 24 different CPR artifact samples from pig data to create CPR-contaminated ECGs with different SNR levels. The SE and SP of the classification on unfiltered data with noise level of −3 dB were 99.7 and 98.6%, respectively. Using their filter method, the SE and SP values of 99.5 and 100% were found. 

In 2019 [23], the same research group introduced a new algorithm to directly analyze CPR-contaminated ECG data to perform shock/no-shock classification. Table 5 in the Results section shows the results of the combined methods on a same independent dataset. There is a big difference between the results of the BP-based algorithm on our test data set, as SE of 88% and SP of 91.3% (for SNR level of −3 dB), and that reported in [23]. There are several possible reasons for this difference. First, the test dataset used in [23] did not have much variety in the rhythms. Our test dataset contained various types of non-shockable rhythms including NSR, supra VT, rapid supra VT, sinus bradycardia, AFib, AFL, heart block, and PVCs, which the other study did not. Second, the other study’s CPR artifacts were gathered from swine. Third, and most importantly, the trained model was not fully tested using an independent test dataset. Therefore, it is likely that the results reported in their study were biased to the specific subjects’ data which led to higher SE and SP values. 

Recently, methods have been introduced based on deep learning approaches to directly analyze CPR-contaminated ECG rhythms without filtering the CPR artifact [17,28]. These algorithms provide a relatively accurate shock versus no-shock classification. However, they require significant training data and computational time of which the latter will require hardware modifications in the current AED devices. The proposed filtering algorithm, because it is computationally simple, can be easily embedded into current AEDs to provide a reliable shock/no-shock decision by the AED’s rhythm classification algorithm. Moreover, the proposed filtering algorithm provides the capability of restoring corrupted shockable and non-shockable ECG data, which may be later used for other clinical applications. 

### Limitations

Several limitations need to be acknowledged and addressed for future studies. First, in this study, the chosen length of the ECG data is 14 s and it was assumed that for this duration the chest compression rate stayed constant. We chose a 14 s data length as this was the maximum required length for the Defibtech AED’s classification algorithm (which was used here to evaluate the proposed method) for a shock/no-shock decision. However, our filtering algorithm also works for a segment as low as 8 s length. Fortunately, we observed that CPR chest compression rates do not deviate significantly in the 14 s interval. Second, we assume that the CPR signal is periodic or quasi-periodic, which led to our approach to examine the fundamental and harmonic frequencies. This was a valid assumption, however, as shown in our results. Third, the proposed algorithm does not suppress greater than 3rd-order harmonics but this did not negatively affect the performance, as the magnitude values of these higher harmonics are inconsequential. However, in order to increase the efficiency of the algorithm in higher SNR levels (−9 dB) further investigations is required. Fourth, removing CPR artifacts from asystole data is a challenging problem. This is because in order to achieve the underlying flatline characteristics of asystole, all CPR-related frequency components need to be suppressed (in addition to the first and second peak frequencies). We only had 52 examples of CPR-contaminated asystole data. Therefore, we were not able to investigate and improve the performance of the proposed filtering algorithm on asystole rhythms. However, multiple representative examples of suppressing both first and second peak frequencies on CPR-contaminated asystole (comprised of successful and failed filtering results) are demonstrated in the Figure S1. Fifth, although we were able to create separate ECG recordings with CPR artifacts as a validation dataset, the performance of the proposed filtering algorithm needs to be further evaluated on real-life ECG data from AED devices during CPR. This is because the source of the CPR-related artifact during CCs has not yet been well addressed and CPR may have more than just an additive effect of CCs on underlying ECG. We hope to address this issue in our future work.

## 5. Conclusions

Analyzing CPR-contaminated ECG signals may lead to erroneous shock decisions by an AED. This work introduced a computationally simple and yet efficient ECG filtering approach which can be used to determine shock versus no-shock decisions while CPR is performed without stoppage. Our method does not require a reference signal and solely uses only 14 s ECG signal segments. Since the algorithm is efficient and real-time realizable, it can be easily implemented on current AED devices without any hardware modification or additional reference signal requirements. Two different databases were used to design and test the algorithm. The SNR-improvement and correlation coefficient metrics indicated that our condition-based filtering algorithm is effective in providing accurate shockable and non-shockable decision via a separate classification algorithm. The SE for CPR-contaminated VF and rapid VT detection was only 67 and 62.7%, applying Defibtech’s rhythm analysis algorithm when no filter was applied, but these values increased to 91.3 and 78%, respectively, with our designed filter. The obtained values meet the AHA’s SE requirement (SE> 90% for VF and > 70% for rapid VT). Although the results are nearly at the AHA threshold of SP, it did not quite meet the requirements. The recently introduced machine learning BP-based classification algorithm was also implemented and tested. The SE and SP of this BP-based method for different corruption levels were 86.3 and 87.8, respectively. When our filter and the BP-based classification algorithm were combined, the SE and SP increased to 94.5 and 88.3%, respectively. Hence, the proposed condition-based filtering algorithm is able to improve the classification results and provide more reliable shockable versus non-shockable decisions for existing AED rhythm analysis algorithms by prefiltering the ECG. However, due to the data unavailability we could not perform enough investigation and provide improvement on asystole cases. We hope to solve this issue in our future work.

## Figures and Tables

**Table 1:** Percentage of high correlation coefficient values between PSD of the artifact-free ECG and the corresponding corrupted and filtered signals.

Rhythm Type	% of Correlation Coefficient > 0.7 between Artifact-Free and CPR-Contaminated ECG	% of Correlation Coefficient > 0.7 between Artifact-Free and ECG after Removing Artifacts’ Highest Frequency Peaks	% of Correlation Coefficient > 0.7 between Artifact-Free and ECG after Removing Artifacts’ 1st and 2nd Frequency Peaks
Non-shockable	18.1%	64%	75.4%
Shockable	18.5%	77.7%	66.5%

**Table 2:** Estimated power (watt/Hz) in the development samples for frequencies within (10–15) Hz.

Rhythm Type	Power within (10–15) Hz (Average ± std)
Shockable	0.027 ± 0.022
Non-shockable	0.084 ± 0.047

**Table 3:** Estimated power (watt/Hz) in the validation samples for frequencies within (10–15) Hz.

Rhythm Type	Power within (10–15) Hz (Average ± std)
Shockable	0.037 ± 0.028
Non-shockable	0.120 ± 0.059

**Table 4:** Results of the proposed filtering algorithm on the validation set.

Rhythm Type	AHA’s Goal	SNR Improvement after Applying Proposed Algorithm	% of Highly Correlated Samples with Desired Clean ECGs before-after Applying Proposed Algorithm	Classification Performance before Applying Proposed Algorithm	Classification Performance after Applying Proposed Algorithm
Shockable					
Coarse VF	>90%	4.1 ± 2.4 dB	8.1–83.5%	67.7%	91.3%
Rapid VT	>75%	3.7 ± 2.6 dB	20–80%	62.7%	78%
Non-shockable					
NSR	>99%	4.8 ± 2.5 dB	9–70%	96.2%	96.5%
Other non-shockables	>95%	4.5 ± 2.5 dB	15.7–69%	91.5%	92.7%

**Table 5:** Averaged results on different SNR levels.

Case	SNRs	Method	SE	SP	ACC
1	[0, −3, −6, −9]	Defibtech classification algorithm	62.1%	88.3%	84.3%
2	[0, −3, −6, −9]	Proposed filtering method + Defibtech classification algorithm	85.5%	89.6%	88.8%
3	[0, −3, −6, −9]	Machine learning (BP)	86.3%	87.8%	87.6%
4	[0, −3, −6, −9]	Proposed filtering method + machine learning (BP)	94.5%	88.3%	89.2%

## Data Availability

Data used in this study are from two sources: publicly available database of PhysioNet Physio-bank archive, and data that was obtained from Defibtech LLC.

## Supplementary Materials

The following are available online at https://www.mdpi.com/article/10.3390/s21248210/s1, Figure S1: Applying the proposed condition-based filtering approach on representative examples asystole rhythms during CPR.
