# Supplementary material for: Automated Condition-Based Suppression of the CPR Artifact in ECG Data to Make a Reliable Shock Decision for AEDs during CPR
Source: Sensors (Basel). 2021 Dec 8;21(24):8210. doi: 10.3390/s21248210 (PMC8708115; doi:10.3390/s21248210)
Supplement: Supplementary file 1 [file sensors-21-08210-s001.zip › sensors-1409053-supplementary.pdf]

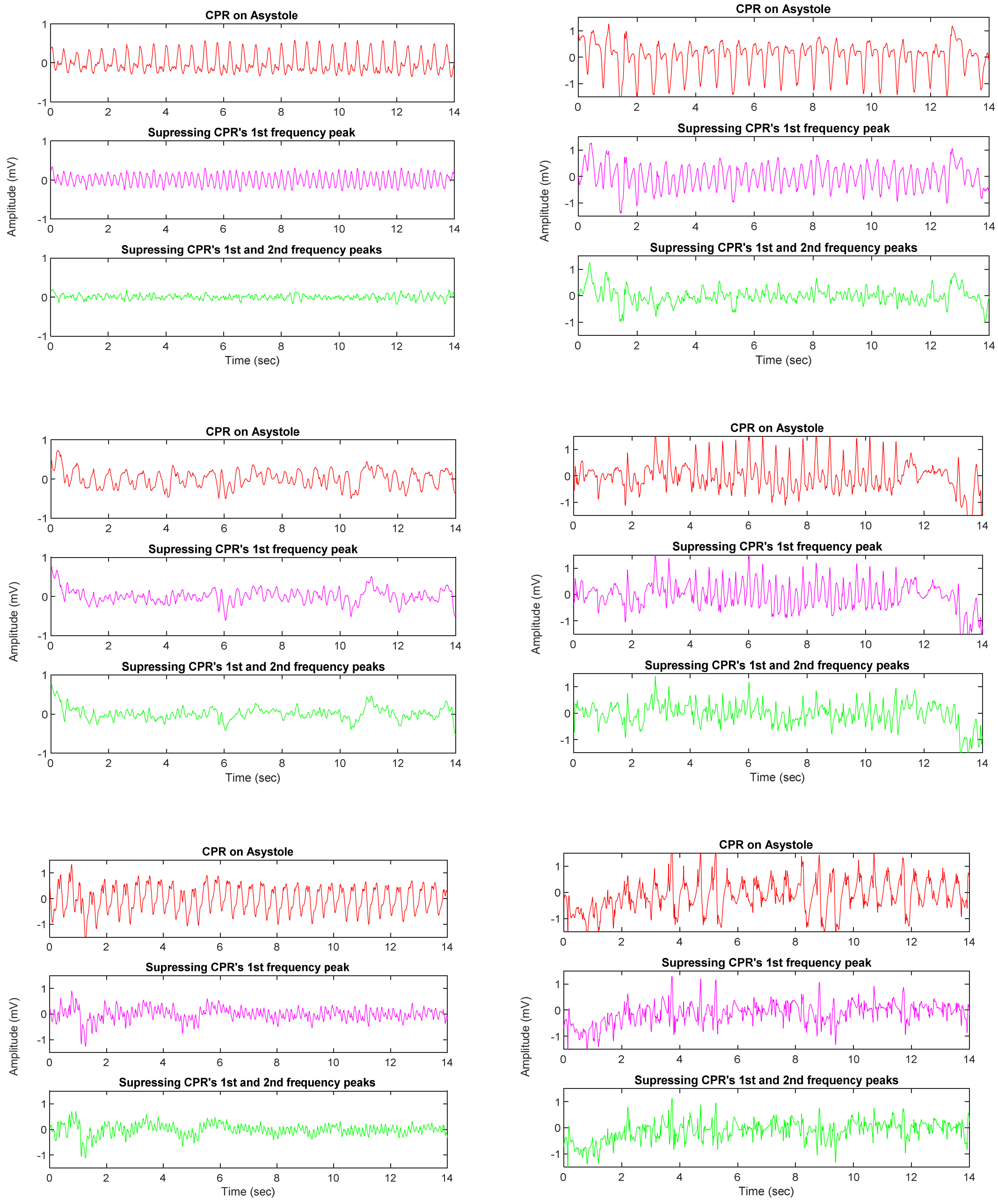

Figure S1. Applying the proposed condition-based filtering approach on representative examples asystole rhythms during CPR. Left and right columns show successful and failed filtering, respectively.
